# LTe2 induces cell apoptosis in multiple myeloma by suppressing AKT phosphorylation at Thr308 and Ser473

**DOI:** 10.3389/fonc.2023.1269670

**Published:** 2023-09-14

**Authors:** Yuanjiao Zhang, Jiacheng Qian, Mingmei Jiang, Shu Yang, Lianxin Zhou, Qin Zhang, Liping Lin, Ye Yang

**Affiliations:** ^1^ Nanjing Hospital of Chinese Medicine and School of Medicine & Holistic Integrative Medicine, Nanjing University of Chinese Medicine, Nanjing, China; ^2^ State Key Laboratory Cultivation Base for TCM Quality and Efficacy, Nanjing University of Chinese Medicine, Nanjing, China; ^3^ Department of Gynecology, Jiangsu Province Hospital Affiliated Hospital of Nanjing Unviersity of Chinese Medicina, Nanjing, China

**Keywords:** multiple myeloma, indole oligomer, LTe2, Akt, phosphorylation, apoptosis

## Abstract

Multiple myeloma (MM) is a highly heterogeneous hematological malignancy originating from B lymphocytes, with a high recurrence rate primarily due to drug resistance. 2-((1H-indol-3-yl)methyl)-3-((3-((1H-indol-3-yl)methyl)-1H-indol-2-yl)methyl)-1H-indole (LTe2), a tetrameric indole oligomer, possesses a wide range of anticancer activities through various mechanisms. Here, we aim to explore the anti-tumor efficiency and potential downstream targets of LTe2 in MM. Its bioactivity was assessed by employing MTT assays, flow cytometry, and the 5TMM3VT mouse model. Additionally, transcriptomic RNA-seq analysis and molecular dynamics (MD) experiments were conducted to elucidate the mechanism underlying LTe2 induced MM cell apoptosis. The results demonstrated that LTe2 significantly inhibited MM cell proliferation both *in vitro* and *in vivo*, and revealed that LTe2 exerts its effect by inhibiting the phosphorylation of AKT at the Thr308 and Ser473 sites. In summary, our findings highlight the potential of LTe2 as a novel candidate drug for MM treatment and provided a solid foundation for future clinical trials involving LTe2.

## 1 Introduction

Multiple Myeloma (MM), the second most common hematological malignancy accounting for ~10% of all hematological malignancies, is a malignant hematological tumor derived from plasma cell, which predominantly affects individuals aged 45 and above (1, 2). The disease is characterized by the presence of abnormal clonal plasma cell in bone marrow, uncontrolled proliferation of MM cells, and the development of destructive bone lesions, kidney damage, anemia and hypercalcemia (3, 4). With the global population aging, the incidence and mortality rates of MM have been increasing steadily (5). Despite the introduction of novel drugs, such as proteasome inhibitors represented by Bortezomib (BTZ), and advancements in treatment approaches like CAR-T therapy, MM remains an incurable disease (6, 7). Therefore, the development of new effective drugs is needed to explore more effective therapeutic strategies to improve the prognosis of patients.

Indole-3-carbinol (I3C), a plant-based small molecule compound, has been shown to modulate a variety of biological processes in mammals and has shown cancer preventive effects in different models (8–10), however its role in MM is unclear. I3C is chemically unstable in acidic aqueous media and microbial cultures and can form structurally distinct compounds (11). In our previous study, we have described how I3C obtained from cruciferous vegetables (i.e., the raw material for kimchi), can be oligomerization in acidic environments, forming 3,3′-diindolylmethane (DIM) and 2-(indol-3-ylmethyl)-3,3′-diindolylmethane (LTr1) (12). As two major I3C metabolites, both DIM and LTr1 have been shown to possess excellent anti-tumor efficacy, and more importantly, DIM has been incorporated into commercial nutraceutical products with anticancer potential (13, 14). Importantly, we further have identified I3C-derived tetramers, pentamers and hexamers from commercially available kimchi products. Interestingly, We have found that the tetramer, 2-((1H-indol-3-yl)methyl)-3-((3-((1H-indol-3-yl)methyl)-1H-indol-2-yl)methyl)-1H-indole (LTe2) could inhibit the proliferation of human leukemia cell lines MV4-11 and THP-1 (15). These studies support our speculations that IC3 and IC3-derived indole oligomers may inhibit MM cell proliferation and exert antitumor effects. Therefore, the present study aimed to further explore the downstream targets and potential mechanisms of various indole oligomers in MM.

We examined the antitumor effects of IC3 and various indole oligomers derived from it on MM and found that the tetramer LTe2 inhibited the proliferation of MM cells *in vitro* and *in vivo* as an effective and safe anti-cancer drug with broad therapeutic applications. Our previous studies have demonstrated the crucial role of the AKT signaling pathway, specifically phosphorylation at the Thr308 and Ser473 sites of AKT, in the development and progression of MM (16, 17). Therefore, these discoveries suggested that LTe2 has the potential to be utilized as a therapeutic agent for suppressing the development and progression of MM.

## 2 Materials and methods

### 2.1 Antibodies and reagents

All primary antibodies were diluted at a ratio of 1:1000 as follows: PARP (9542S, Cell Signaling Technology), Caspase-3 (19677-1-AP, Proteintech), Cleaved Caspase-3 (9661S, Cell Signaling Technology), β-actin (60008-1-Ig, Proteintech), CDK4 (11026-1-AP, Proteintech), CDK6 (14052-1-AP, Proteintech), CyclinD1 (55506T, Cell Signaling Technology), AKT (9272s, Cell Signaling Technology), P-AKT Thr308 (9275s, Cell Signaling Technology), P-AKT Ser473 (4060S, Cell Signaling Technology). The second antibodies were diluted 1:5000 and purchased from Santa cruz (California, USA), including goat anti-Rabbit IgG (sc-2005) and rabbit anti-mouse IgG (sc-2004).

### 2.2 Cell lines and culture

Human MM cell lines (ARP1, CAG, H929, KMS28BM, OCI-MY5), mouse MM cell line 5TMM3VT cells and human colorectal cancer (CRC) cell lines (RKO, CW-2) were cultured in RPMI-1640 (C3010-0500, VivaCell). The culture medium was supplemented with 10% fetal bovine serum (A6901FBS-500, Invigentech, California, USA), and 1% penicillin/streptomycin (C100C5, NCM Biotech, Suzhou, China). All cells were cultured in an incubator at a temperature of 37°C and a CO_2_ concentration of 5%.

### 2.3 Cell proliferation

Cells (6 × 10^3^/well) were seeded in a 96-well plate with a cell suspension volume of 180 μL/well, and then 20 μL of different concentrations of drugs were added to each well and incubated for the specified duration. MTT (3-(4,5-dimethylthiazol-2-yl)-2,5-diphenyltetrazolium bromide) assay was performed to test cell viability and proliferation. After the incubation period, 20 μL of MTT reagent (5 mg/mL) was added to each well, and the plate was incubated for an additional 4 h. The MTT formazan crystals formed by metabolically active cells were solubilized. The absorbance was measured spectrophotometrically at 570 nm by using a microplate reader (Thermo Fisher Scientific, USA).

### 2.4 Cell cycle and apoptosis assays

5 μL of propidium iodide (PI) solution (No. C0080, Solarbio) was added to detect the cell cycle. 5 μL of APC Annexin V (640941, Biolegend, California, USA) and 5 μL of PI (No. C0080, Solarbio, Shanghai, China) were added to detect the cell apoptosis. The cell samples were analyzed by flow cytometry (Merck Millipore, Germany).

### 2.5 Western blotting

The total protein was extracted by RIPA lysis buffer and quantified by BCA Protein Quantification Kit (20201ES86, YEASEN) using a microplate reader (Thermo Fisher Scientific, USA). A total of 20 μg of protein was resolved by SDS-PAGE and transferred onto a 0.45 μm PVDF membrane (Millipore, Bedford, MA). After blocking the membrane with 5% non-fat milk or BSA for 1 h, it was incubated with primary antibodies overnight, followed by incubation with secondary antibodies for 1 h. Using super ECL Detection Reagent (36208ES60, YEASEN) to visualize.

### 2.6 Transcriptomic RNA-sequencing

F2, LTe2, DIM, I3C were added to ARP1 WT cells for treatment and then performed mRNA sequencing. All data analysis and processing are conducted by Lc-Bio Technologies (Hangzhou) Co., Ltd. (Hangzhou, Zhejiang, China) and deposited in NCBI GEO database (accession number GSE189309).

### 2.7 5TMM3VT mouse model

5TMM3VT cells (1 × 10^6^) were administered via tail intravenous (i.v.) injection in 6-week-old male C57BL/KaLwRij mice (n = 10 per group). After 2 days the mice were randomly divided into two groups: the control group and the treatment group. The control group received injections of physiological saline, while the treatment group received injections of LTe2 at a dosage of 50 mg/kg twice a week. The injections were continued until the mice were either sacrificed or died. The survival time of each group of mice was recorded, and the data were used to plot survival curves (18).

### 2.8 Statistical analysis

Data were presented as the mean ± standard deviation and analyzed by using a two-tailed Student’s t-test (2 groups). Statistical analyses were performed using SPSS version 19.0 and Prism 8.0 software. *p*<0.05 (*), *p*<0.01 (**) and *p*<0.001 (***) were considered to indicate statistically significant differences.

## 3 Results

### 3.1 LTe2, tetramer of I3C, is screened out as the most potent inhibitory activity from various indole oligomers

**Figure 1:**
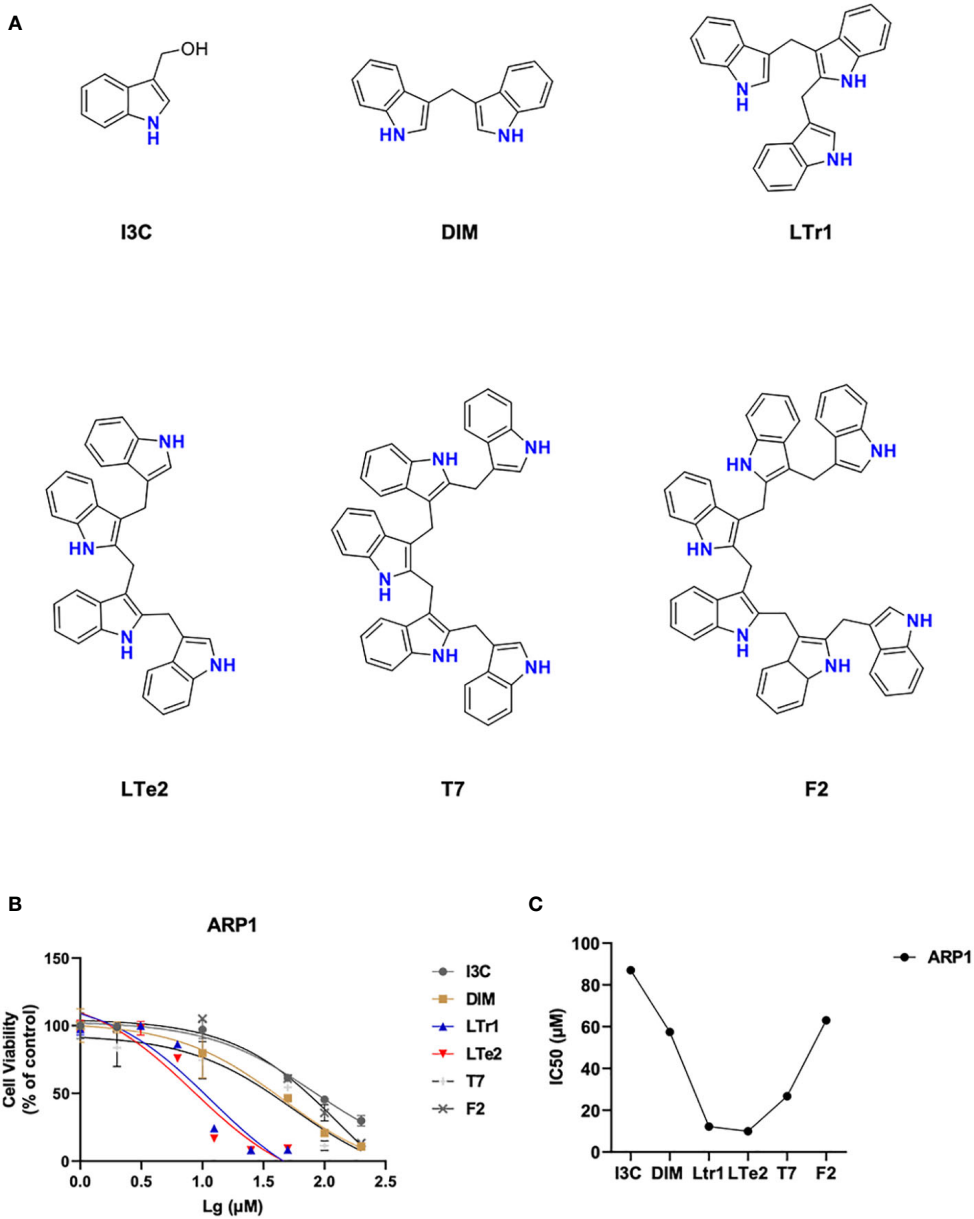
LTe2, tetramer of I3C, is screened out as the most potent inhibitory activity from various indole oligomers. **(A)** The chemical structure of I3C, DIM, LTr1, LTe2, T7 and F2. **(B, C)** Effects of I3C, DIM, LTr1, LTe2, T7 and F2 on viability of ARP1 WT cells.

To compare the effects of various indole oligomers on ARP1 cell activity, I3C (monomers), DIM (dimers), LTr1 (trimers), LTe2 (tetramers), T7 (pentamers) and F2 (hexamers) were assayed by using MTT method. The structures of these various indole oligomers depicted in **Figure 1A**. The results indicated that LTe2 (tetramers) exhibited the most potent inhibitory effect on the proliferation of MM cells (**Figures 1B, C**). These findings provided preliminary evidence supporting the potential of LTe2 targeted inhibiting of MM cell proliferation.

### 3.2 LTe2 inhibits cellular proliferation in MM cell lines

**Figure 2:**
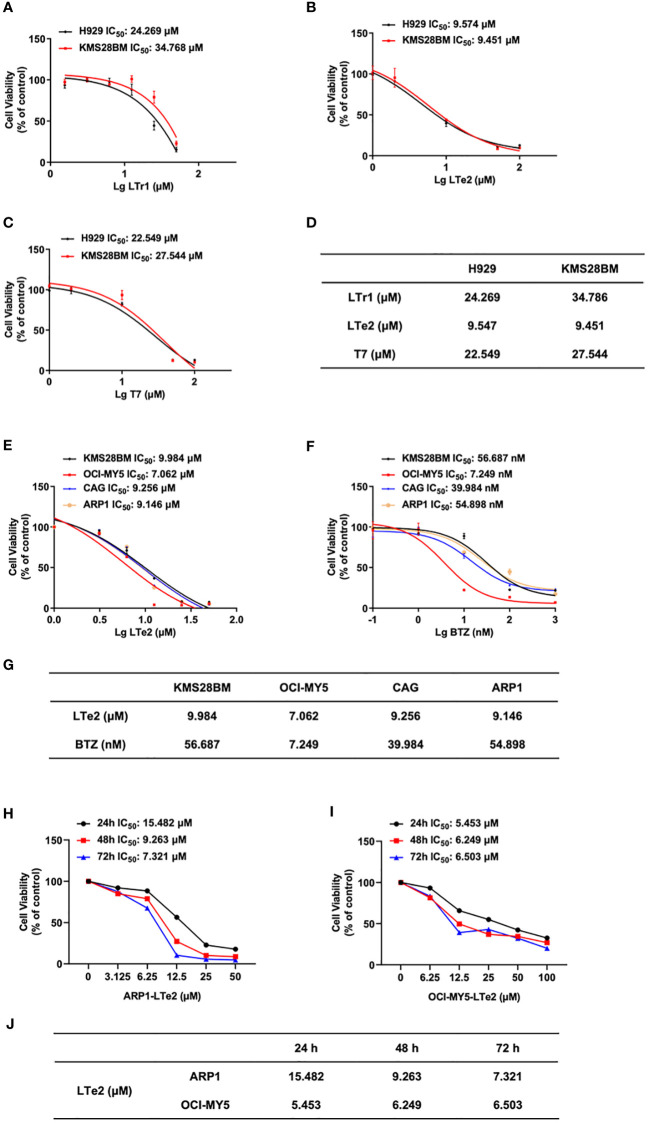
LTe2 inhibits cellular proliferation in MM cell lines. **(A–D)** Effects of LTe2, T7 and LTr1 on viability of H929 WT and KMS28BM WT cells. **(E–G)** Effects of LTe2 and BTZ on viability of KMS28BM WT, OCI-MY5 WT, CAG WT and ARP1 WT cells. **(H–J)** Effects of LTe2 on viability of ARP1 WT and OCI-MY5 WT cells at 24 h, 48 h and 72 h.

To further evaluate the potential antitumor activity of indole oligomers in different type of cancers, we conducted an MTT assay using a series of indole oligomers on colorectal cancer (CRC) cells (**Supplementary Figure S1**) as well as MM cells. Among the compounds tested, we found that indole oligomers inhibited MM cells better than CRC cells, with LTe2, T7, and LTr1 showing the best inhibitory activity on MM cell viability following a screening process. Subsequently, we further employed the MTT method to determine the IC_50_ values of LTe2, T7, LTr1 in two MM cells, respectively. Our findings revealed that LTe2 (IC_50-H929 =_ 9.547 μM, IC_50-KMS28BM_=9.451 μM) demonstrated a promising anti-proliferation activity in both MM cells, exhibiting the lowest IC_50_ values among the tested compounds (**Figures 2A–C**). And the specific IC_50_ values were presented in the table (**Figure 2D**). In addition, IC_50_ values of LTe2 and conventional chemotherapeutic drugs for MM treatment, BTZ, in four types of MM cells were compared using the MTT assay (**Figures 2E, F**). Based on the IC_50_ values (**Figure 2G**), we further selected ARP1 (IC_50-ARP1 =_ 9.146 μM) and OCI-MY5 (IC_50-OCI-MY5 =_ 7.062 μM) cells with higher activity concentrations when treated with LTe2 for further study. The results showed LTe2 exhibited a promising anti-proliferation activity within a three-day period in the selected cells (**Figures 2H, I**). The specific IC_50_ values were presented in the table (**Figure 2J**). Collectively, these findings strongly suggest that LTe2 possesses promising inhibitory activity against MM cell proliferation.

### 3.3 LTe2 inhibits proliferation and induces apoptosis of MM cells

**Figure 3:**
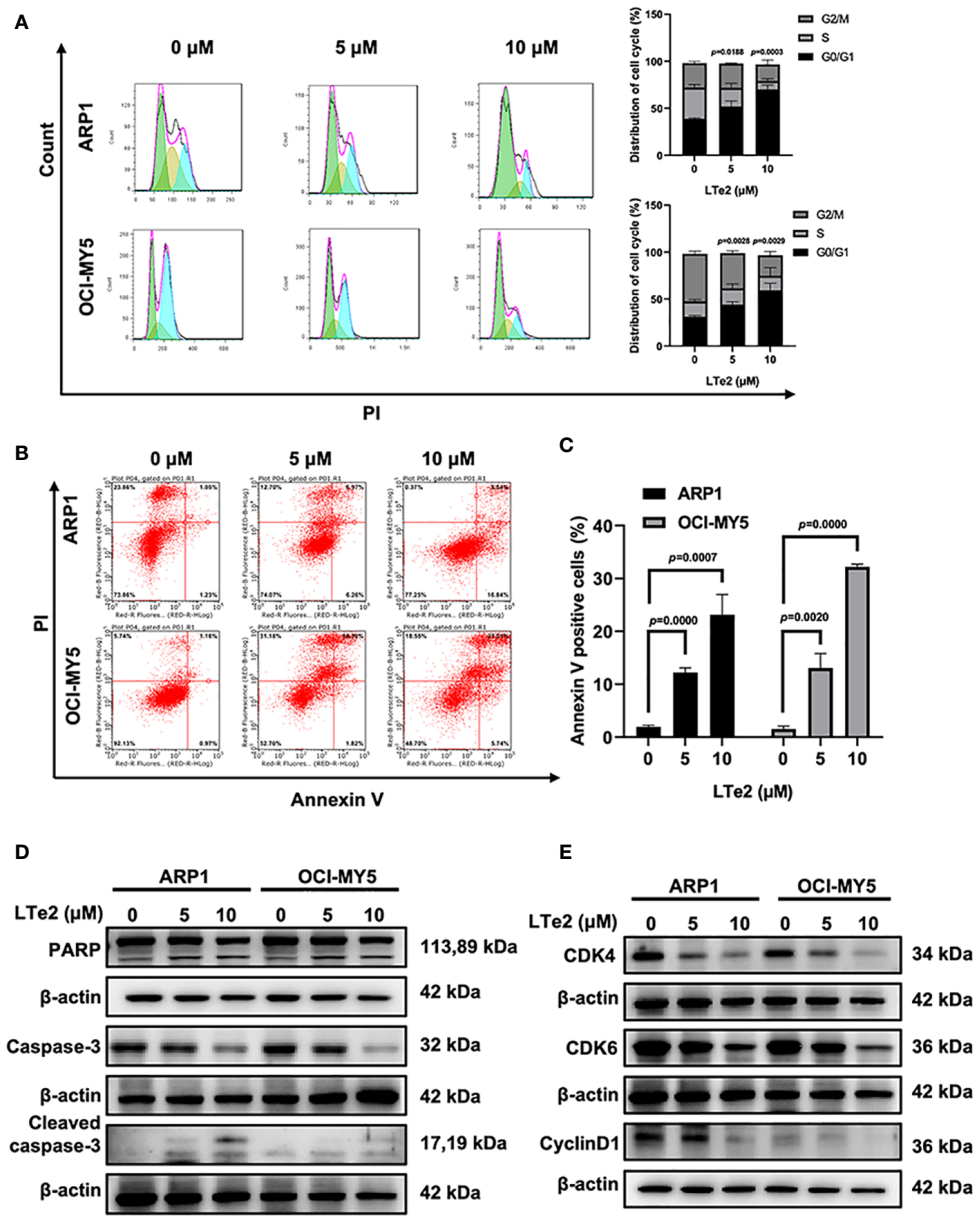
LTe2 inhibits proliferation and induces apoptosis of MM cells. **(A)** Flow cytometry analysis displayed that treatment with LTe2 increased G0/G1 phase fraction in ARP1 WT and OCI-MY5 WT cells. **(B)** Flow cytometry analysis indicated that LTe2 facilitated cell apoptosis of ARP1 WT and OCI-MY5 WT cells. **(C)** The apoptotic rate of ARP1 WT and OCI-MY5 WT cells after treatment with LTe2 was determined quantitatively as histograms. **(D)** WB analysis confirmed that LTe2 increased the expressions of apoptotic proteins: caspase-3 & cleaved caspase-3 and PARP & cleaved PARP. **(E)** WB assays indicted that treatment with LTe2 decreased CDK4, CDK6 and CyclinD1 protein expressions in MM cells. The data are expressed as mean ± SD.

To confirm whether LTe2 inhibited cells by affecting cell cycle, we next employed flow cytometry analysis to examine cell cycle, showing that treatment with LTe2 arrested the proportion of cells in the G0/G1 phase (*p*=0.0188, *p*=0.0003, *p*=0.0028, *p*=0.0029) (**Figure 3A**). As apoptosis is closely related to cell cycle in cancer cells, we followed to utilize Annexin V/PI staining assay to show that with the increase of LTe2 concentration, the ability to promote apoptosis of MM cells was enhanced (*p*=0.0000, *p=*0.0007, *p=*0.0020, *p*=0.0000) (**Figures 3B, C**). Consistently, the results of WB confirmed the increased expression of apoptosis markers: the cleaved PARP (poly ADP-ribose polymerase) as well as cleaved caspase-3 after LTe2 treatments (**Figure 3D**; **Supplementary Figure S2**), and the protein expressions of CDK4, CDK6 and CyclinD1 were decreased upon LTe2 treatment (**Figure 3E**; **Supplementary Figure S3**). Collectively, the above data suggest that LTe2 inhibits the proliferation and induces apoptosis of MM cells *in vitro*.

### 3.4 LTe2 extends the survival of 5TMM3VT MM mice

**Figure 4:**
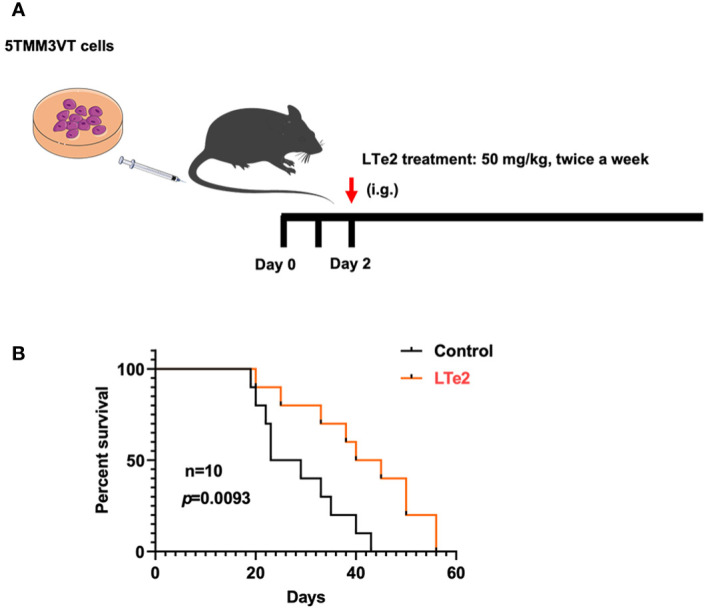
LTe2 extends the survival of 5TMM3VT MM mice. **(A)** Schematic of 5TMM3VT mouse model. **(B)** LTe2 greatly improved the survival time of MM-prone C57BL/KaLwRij mice (n = 10).

To verify the inhibitory effects of LTe2 on MM progression *in vivo*, we constructed a 5TMM3VT MM mouse model, which could be a mature and generalized MM mouse model *in vivo *(19, 20), as illustrated in **Figure 4A**. The LTe2 treatment group (50 mg/kg body weight, twice a week) exhibited a significant extension in the survival of myeloma mice compared to the control group (*p*=0.0093) (**Figure 4B**). These findings indicate that LTe2 prolongs the survival of 5TMM3VT mice *in vivo* and possesses potent anti-tumor activity.

### 3.5 The activity of various indole oligomers is associated with apoptosis and AKT signaling pathways and LTe2 targeting is optimal

**Figure 5:**
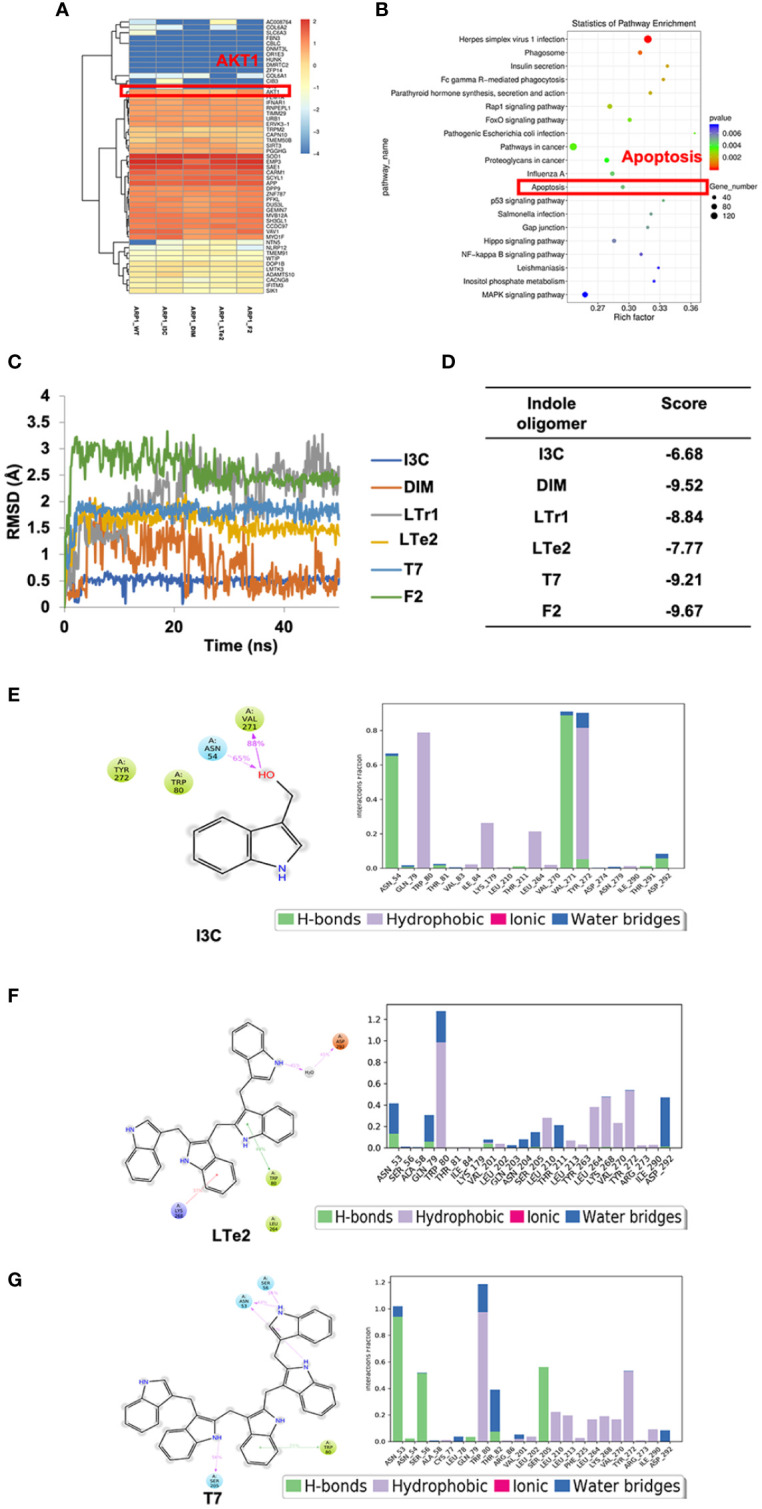
The activity of various indole oligomers is associated with apoptosis and AKT signaling pathways and LTe2 targeting is optimal. **(A)** The heat map showed that AKT1 was considered a differentially expressed gene after treatment of indole oligomers. **(B)** KEGG pathway analysis of the RNA-seq data indicated that various indole oligomers were associated with the apoptosis signaling pathway. **(C)** The molecular dynamics (MD) experiments showed that I3C, LTe2 and T7 have relatively low RMSD and stable binding mode. **(D)** The score value of molecular docking. **(E–G)** The combination modes of I3C, LTe2 and T7.

To further investigate the mechanism underlying the inhibitory effect of LTe2 on MM cell proliferation, we performed transcriptomic RNA-seq on ARP1 cells treated with I3C (monomers), DIM (dimers), LTe2 (tetramers), and F2 (hexamers) or not as a control. Based on the similarity of the gene expression profiles of the samples, we performed clustering analysis of the differentially expressed genes (DEG) to visualize the expression of the genes in different treatments, and the heatmap showed that the AKT1 gene was highly expressed in all groups (**Figure 5A**). Next, we performed Kyoto Encyclopedia of Genes and Genomes (KEGG) pathway enrichment analysis on the DEG, which were closely associated with apoptosis signaling pathway after treatment of MM cells with various indole polymers (**Figure 5B**; **Supplementary table S1**). Additionally, further investigation through the KEGG pathway revealed that the AKT signaling pathway is also one of the classic cell apoptosis pathways. To further confirm the binding affinity between LTe2 and the phosphorylation site of AKT1, we initially scored it through MD experiment. The MD experiment results showed that after a 50ns MD, DIM and LTr1 remained in an unstable state (**Figure 5C**). The RMSD value of compound F2 was the largest, indicating a significant deviation from the initial binding mode. On the other hand, compounds I3C, LTe2 and T7 exhibited relatively low RMSD values and stable binding modes. Especially the RMSD value of I3C is the lowest (**Figure 5D**). Furthermore, we analyzed and compared the combination modes of I3C, LTe2 and T7 and found that W80 might interact with the indole ring to form pi-pi bonds. IC3 had a simple structure and exerted a single force, resulting in relatively weak binding ability (**Figure 5E**). LTe2 tended to form hydrogen/ion bonds with D292 or a hydrogen bonding network through water molecules as bridges (**Figure 5F**). However, due to factors such as molecular volume, T7 did not easily form hydrogen bonds with the receptor protein D292 (**Figure 5G**). Based on these findings, we speculate that LTe2 could inhibit MM cell proliferation by targeting AKT phosphorylation.

### 3.6 LTe2 promotes apoptosis of MM cells through inhibition of AKT phosphorylation at Thr308 and Ser473

**Figure 6:**
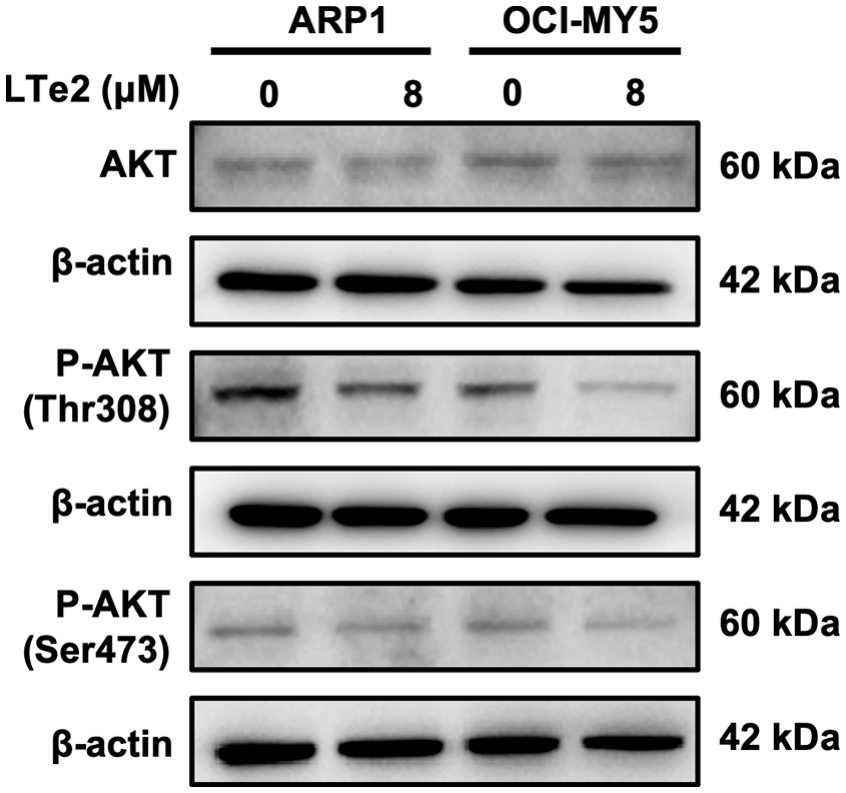
LTe2 promotes apoptosis of MM cells through inhibition of AKT phosphorylation at Thr308 and Ser473. WB validated the protein expression profiles of AKT, P-AKT (Thr308) and P-AKT (Ser473) in ARP1 WT and OCI-MY5 WT cells with or without treatment of LTe2.

**Figure 7:**
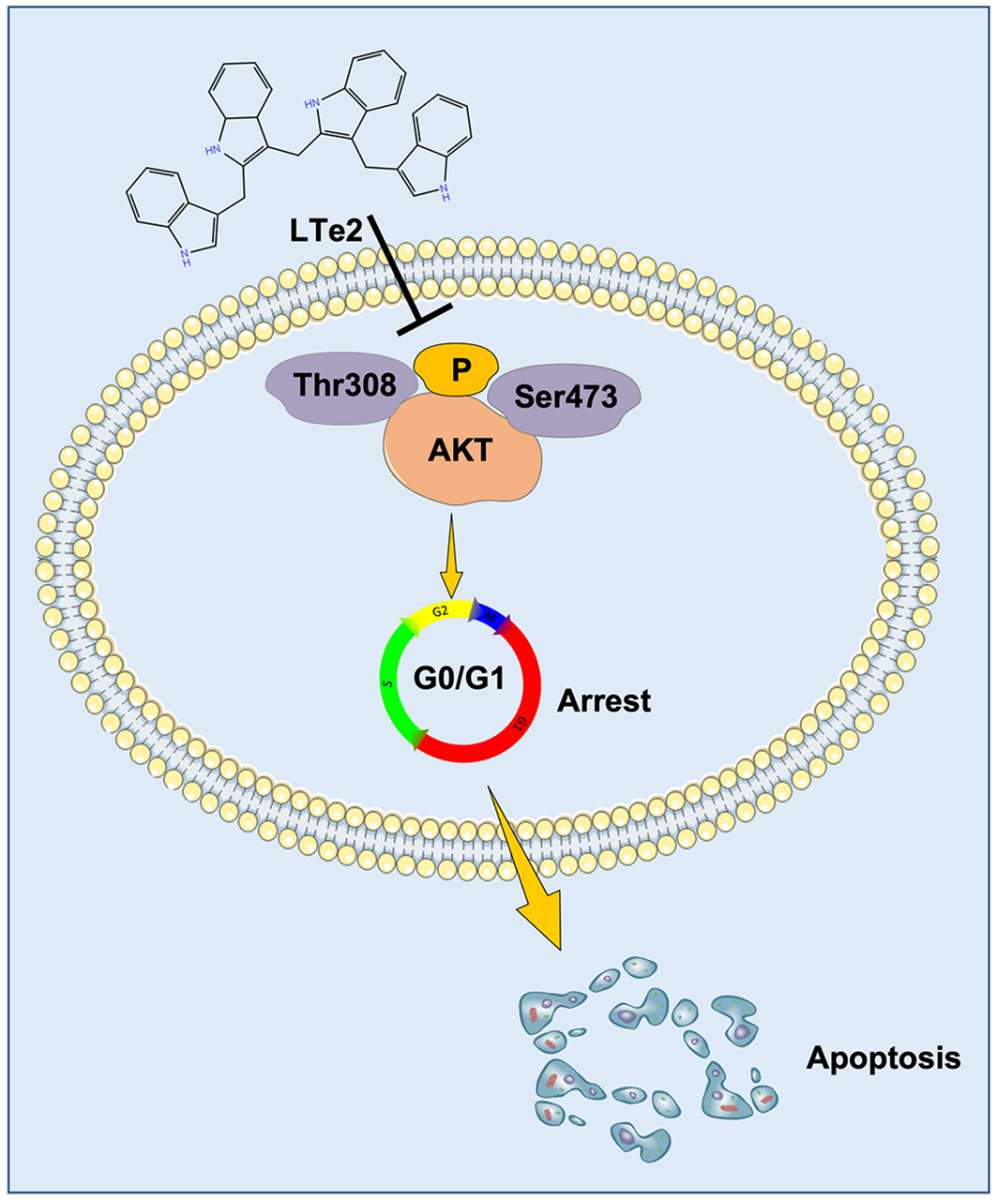
Schematic diagram of the mechanism of LTe2 promoting apoptosis of MM cells.

Due to complete activation of AKT requires the phosphorylation at Thr308 and Ser473 sites (21), AKT activation plays a crucial role in various cellular processes, including cell proliferation and apoptosis (22). To confirm the involvement of LTe2 in regulating AKT phosphorylation, we examined the expression of AKT, P-AKT (Thr308), and P-AKT (Ser473) in MM cells with or without LTe2 treatment. Western blot results confirmed that LTe2 treatment substantially downregulated the expression of P-AKT (Thr308) and P-AKT (Ser473) in both ARP1 and OCI-MY5 cell lines (**Figure 6**; **Supplementary Figure S4**). In summary, LTe2 decreased the phosphorylation of AKT at the Thr308 and Ser473 sites. These findings suggest that LTe2 inhibits the proliferation of MM cells via targeting AKT phosphorylation, making it a potential effective drug for MM patients (**Figure 7**).

## 4 Discussion

Protein Posttranslational Modifications (PTMs) and related drugs play a very important role in the pathogenesis of MM and drug development. Proteasome inhibition is an effective and commonly used treatment in MM, and drugs such as bortezomib, carfilzomib, and ixazomib have been approved for the clinical treatment of MM (23, 24). Inhibition of the proteasome leads to proteotoxic and genotoxic stress that drives apoptosis in MM cells (25), which suggests that PTM may be an important factor in the development of MM. PTMs play a significant role in various biological processes by regulating enzyme activity, protein stability, interaction and so on (26). The importance of PTM has attracted increasing attention, and > 450 unique PTMs have been discovered so far, including phosphorylation, acetylation, and ubiquitination. Dysregulation of PTM has been implicated in the development of diseases such as cancer (27, 28). Thus, we believe that the solution to MM resistance and malignant proliferation or the lack of new drugs should start from PTM. In this paper, we found that the natural small molecule compound IC3 is a good antitumor drug, and previous studies have shown that indole oligomers have significant pleiotropic effects on cancer cells by inactivating survival signals and activating multiple cell death pathways simultaneously (13, 29). However, the effect of IC3 on MM is still unclear. In particular, our data suggested that IC3 inhibit the proliferation of MM cells, and more importantly, the linear tetramer LTe2 was effective in inhibiting the growth and inducing apoptosis of MM cells both *in vitro* and *in vivo*. These results highlight the potential of LTe2 treatment in MM cells. During the process of animal experiments, we did observe the general condition of the mice who received LTe2. They looked healthy without obvious symptoms such as vomiting or convulsions, which were not significantly different from the mice not receiving LTe2 treatment. Besides, as an indirect indicator of systemic toxicity, the body weight changes were monitored throughout the experiment. Compared to the control group, the mice in LTe2 group did not show a significant decrease in body weight, reflecting the safety of the LTe2 treatment. Accordingly, we hypothesized that LTe2 may be a specific drug target in MM cells, opening up new therapeutic avenues in anti-myeloma therapies.

Our studies show that the I3C-derived tetramer LTe2 significantly inhibits the growth of MM cells. Kimchi provided an acidic environment for the oligomerization of I3C-derived indoles, which resulted in the derivation of indole dimers to hexamers. From this we know that the Michael addition of 3MI to the indole nucleus is regioselective, a result that can stimulate the construction and/or modification of indole alkaloids (15, 30). The discovery of more and more natural products also provides new inspiration for drug development.

At present, inducing apoptosis has emerged as a promising therapeutic approach for cancer treatment. Therefore, seeking bioactive compounds that can induce apoptosis represents a valuable strategy to inhibit carcinogenesis and prevent further malignant proliferation of tumor cells. Cell cycle dysregulation is a common feature of human cancers, and the cell cycle is closely related to apoptosis (17, 31). Blocking cell cycle progression and inducing apoptosis are two important mechanisms by which antiproliferative genes inhibit cancer cell growth. DIM, in particular, is well-known for its ability to induce cell cycle arrest and apoptosis, making it an effective anticancer agent (32). Therefore, we hypothesized whether the inhibition of MM cells by LTe2 was also related through induced apoptosis and cell cycle arrest. Our data demonstrated that treatment with LTe2 induced cell apoptosis, as evidenced by an increase in the proportion of cells in the G0/G1 phase and a decrease in the expressions of CDK4 and CDK6. These findings further emphasize the close relationship between cell cycle and apoptosis.

Furthermore, in combination with RNA-seq we found that indole oligomers could inhibit MM cell proliferation by suppressing AKT phosphorylation. AKT activation and degradation are known to be involved in various biological responses, such as cell survival, proliferation, protein translation, and metabolism (33–35). Many oncogenes and proto-oncogenes are protein kinases that undergo phosphorylation, and their activation often contributes to tumorigenesis (36, 37). Importantly, phosphorylation is one of the most extensively studied PTMs of AKT, which phosphorylation at Thr308 and Ser473 is essential for AKT activation (38, 39). Intriguingly, our study revealed that LTe2 inhibits the phosphorylation of AKT at the Thr308 and Ser473 sites. We demonstrated that the activation of AKT phosphorylation is associated with the promotion of MM cells, as AKT is a known biomarker for cell survival (40). Although our study has identified the potential of LTe2 as a new drug of MM treatment, one limitation is the incomplete characterization of the direct involvement of the drug-target interaction within the AKT pathway. Further investigations are needed to fully understand the mechanisms underlying the effects of LTe2 in MM cells.

## 5 Conclusions

In conclusion, our findings provide compelling evidence for the significant biological activity of LTe2 in MM treatment. Furthermore, our study has established a valuable strategy for the discovery of novel inhibitors specifically targeting MM. These results hold promise for the development of effective therapeutic approaches and contribute to advancing our understanding of MM treatment.

## Data availability statement

The datasets presented in this study can be found in online repositories. The names of the repository/repositories and accession number(s) can be found in the article/**Supplementary Material**.

## Ethics statement

The animal study was approved by Institutional Ethics Review Boards of Nanjing University of Chinese Medicine (Ethics Registration no. 201905A003). The study was conducted in accordance with the local legislation and institutional requirements.

## Author contributions

YZ: Formal Analysis, Writing – original draft, Conceptualization, Methodology. JQ: Writing – review & editing, Methodology, Resources. MJ: Formal Analysis, Validation, Writing – review & editing. SY: Data curation, Validation, Writing – review & editing. LZ: Methodology, Validation, Writing – review & editing. QZ: Formal Analysis, Resources, Writing – review & editing. LL: Funding acquisition, Writing – review & editing, Methodology, Resources. YY: Investigation, Resources, Writing – review & editing, Funding acquisition, Supervision.

## References

[1] Kumar SK Rajkumar V Kyle RA van Duin M Sonneveld P Mateos MV. Multiple myeloma. Nat Rev Dis Primers (2017) 3:17046. doi: 10.1038/nrdp.2017.46 28726797

[2] Siegel RL Miller KD Fuchs HE Jemal A. Cancer statistics, 2022. CA Cancer J Clin (2022) 72(1):7–33. doi: 10.3322/caac.21708 35020204

[3] Cowan AJ Green DJ Kwok M Lee S Coffey DG Holmberg LA. Diagnosis and management of multiple myeloma: A review. JAMA (2022) 327(5):464–77. doi: 10.1001/jama.2022.0003 35103762

[4] Brigle K Rogers B. Pathobiology and diagnosis of multiple myeloma. Semin Oncol Nurs (2017) 33(3):225–36. doi: 10.1016/j.soncn.2017.05.012 28688533

[5] Mian H Wildes TM Vij R Pianko MJ Major A Fiala MA. Dynamic frailty risk assessment among older adults with multiple myeloma: A population-based cohort study. Blood Cancer J (2023) 13(1):76. doi: 10.1038/s41408-023-00843-5 37164972 PMC10172354

[6] Joshua DE Bryant C Dix C Gibson J Ho J. Biology and therapy of multiple myeloma. Med J Aust (2019) 210(8):375–80. doi: 10.5694/mja2.50129 31012120

[7] Kumar SK Rajkumar SV. The multiple myelomas - current concepts in cytogenetic classification and therapy. Nat Rev Clin Oncol (2018) 15(7):409–21. doi: 10.1038/s41571-018-0018-y 29686421

[8] Wu Y Li RW Huang H Fletcher A Yu L Pham Q. Inhibition of tumor growth by dietary indole-3-carbinol in a prostate cancer xenograft model may be associated with disrupted gut microbial interactions. Nutrients (2019) 11(2):467. doi: 10.3390/nu11020467 30813350 PMC6413210

[9] Lee YR Chen M Lee JD Zhang J Lin SY Fu TM. Reactivation of PTEN tumor suppressor for cancer treatment through inhibition of a MYC-WWP1 inhibitory pathway. Science (2019) 364(6441):eaau0159. doi: 10.1126/science.aau0159 31097636 PMC7081834

[10] Tin AS Park AH Sundar SN Firestone GL. Essential role of the cancer stem/progenitor cell marker nucleostemin for indole-3-carbinol anti-proliferative responsiveness in human breast cancer cells. BMC Biol (2014) 12:72. doi: 10.1186/s12915-014-0072-6 25209720 PMC4180847

[11] Lin LP Yuan P Jiang N Mei YN Zhang WJ Wu HM. Gene-inspired mycosynthesis of skeletally new indole alkaloids. Org Lett (2015) 17(11):2610–3. doi: 10.1021/acs.orglett.5b00882 25985278

[12] Lin LP Liu D Qian JC Wu L Zhao Q Tan RX. Post-ingestion conversion of dietary indoles into anticancer agents. Natl Sci Rev (2022) 9(4):nwab144. doi: 10.1093/nsr/nwab144 35505660 PMC9053945

[13] Banerjee S Kong D Wang Z Bao B Hillman GG Sarkar FH. Attenuation of multi-targeted proliferation-linked signaling by 3,3’-diindolylmethane (DIM): from bench to clinic. Mutat Res (2011) 728(1-2):47–66. doi: 10.1016/j.mrrev.2011.06.001 21703360 PMC4120774

[14] Tian X Liu K Zu X Ma F Li Z Lee M. 3,3’-Diindolylmethane inhibits patient-derived xenograft colon tumor growth by targeting COX1/2 and ERK1/2. Cancer Lett (2019) 448:20–30. doi: 10.1016/j.canlet.2019.01.031 30716361

[15] Cheng Qian J Liu D Ping Lin L Jing Zhu W Xiang Tan R. Minor bioactive indoles from kimchi mirror the regioselectivity in indole-3-carbinol oligomerization. Food Chem (2022) 382:132571. doi: 10.1016/j.foodchem.2022.132571 35245758

[16] Dou R Qian J Wu W Zhang Y Yuan Y Guo M. Suppression of steroid 5alpha-reductase type I promotes cellular apoptosis and autophagy via PI3K/Akt/mTOR pathway in multiple myeloma. Cell Death Dis (2021) 12(2):206. doi: 10.1038/s41419-021-03510-4 33627630 PMC7904855

[17] Zhang Y Deng Z Sun S Xie S Jiang M Chen B. NAT10 acetylates BCL-XL mRNA to promote the proliferation of multiple myeloma cells through PI3K-AKT pathway. Front Oncol (2022) 12:967811. doi: 10.3389/fonc.2022.967811 35978804 PMC9376478

[18] Ke MY Qian JJ Hao F Li XY Wu HJ Luo X. Acupuncture synergized with bortezomib improves survival of multiple myeloma mice via decreasing metabolic ornithine. Front Oncol (2021) 11. doi: 10.3389/fonc.2021.779562 PMC8596548 34804983

[19] Gu C Wang Y Zhang L Qiao L Sun S Shao M. AHSA1 is a promising therapeutic target for cellular proliferation and proteasome inhibitor resistance in multiple myeloma. J Exp Clin Cancer Res (2022) 41(1):11. doi: 10.1186/s13046-021-02220-1 34991674 PMC8734095

[20] Alici E Konstantinidis KV Aints A Dilber MS Abedi-Valugerdi M. Visualization of 5T33 myeloma cells in the C57BL/KaLwRij mouse: establishment of a new syngeneic murine model of multiple myeloma. Exp Hematol (2004) 32(11):1064–72. doi: 10.1016/j.exphem.2004.07.019 15539084

[21] Xiong Y Ju L Yuan L Chen L Wang G Xu H. KNSTRN promotes tumorigenesis and gemcitabine resistance by activating AKT in bladder cancer. Oncogene (2021) 40(9):1595–608. doi: 10.1038/s41388-020-01634-z 33452459

[22] Cheung M Testa JR. Diverse mechanisms of AKT pathway activation in human Malignancy. Curr Cancer Drug Targets (2013) 13(3):234–44. doi: 10.2174/1568009611313030002 PMC3678724 23297823

[23] Dimopoulos MA Merlini G Bridoux F Leung N Mikhael J Harrison SJ. Management of multiple myeloma-related renal impairment: recommendations from the International Myeloma Working Group. Lancet Oncol (2023) 24(7):e293–311. doi: 10.1016/S1470-2045(23)00223-1 37414019

[24] Kegyes D Gulei D Drula R Cenariu D Tigu B Dima D. Proteasome inhibition in combination with immunotherapies: State-of-the-Art in multiple myeloma. Blood Rev (2023) 61:101100. doi: 10.1016/j.blre.2023.101100 37291017

[25] Gandolfi S Laubach JP Hideshima T Chauhan D Anderson KC Richardson PG. The proteasome and proteasome inhibitors in multiple myeloma. Cancer Metastasis Rev (2017) 36(4):561–84. doi: 10.1007/s10555-017-9707-8 29196868

[26] Vu LD Gevaert K De Smet I. Protein language: post-translational modifications talking to each other. Trends Plant Sci (2018) 23(12):1068–80. doi: 10.1016/j.tplants.2018.09.004 30279071

[27] Jarrold J Davies CC. PRMTs and arginine methylation: cancer’s best-kept secret? Trends Mol Med (2019) 25(11):993–1009. doi: 10.1016/j.molmed.2019.05.007 31230909

[28] Deng L Meng T Chen L Wei W Wang P. The role of ubiquitination in tumorigenesis and targeted drug discovery. Signal Transduct Target Ther (2020) 5(1):11. doi: 10.1038/s41392-020-0107-0 32296023 PMC7048745

[29] Lee SO Li X Khan S Safe S. Targeting NR4A1 (TR3) in cancer cells and tumors. Expert Opin Ther Targets (2011) 15(2):195–206. doi: 10.1517/14728222.2011.547481 21204731 PMC4407471

[30] Lin LP Tan RX. Bioactive alkaloids from indole-3-carbinol exposed culture of daldiniaeschscholzii. Chin J Chem (2018) 36(8):749–53. doi: 10.1002/cjoc.201800160

[31] Vermeulen K Berneman ZN Van Bockstaele DR. Cell cycle and apoptosis. Cell Prolif (2003) 36(3):165–75. doi: 10.1046/j.1365-2184.2003.00267.x PMC6496173 12814432

[32] Li Y Li X Guo B. Chemopreventive agent 3,3’-diindolylmethane selectively induces proteasomal degradation of class I histone deacetylases. Cancer Res (2010) 70(2):646–54. doi: 10.1158/0008-5472.CAN-09-1924 PMC2808120 20068155

[33] Liao W Du J Li L Wu X Chen X Feng Q. CircZNF215 promotes tumor growth and metastasis through inactivation of the PTEN/AKT pathway in intrahepatic cholangiocarcinoma. J Exp Clin Cancer Res (2023) 42(1):125. doi: 10.1186/s13046-023-02699-w 37198696 PMC10193609

[34] Park J An G Park H Hong T Lim W Song G. Developmental defects induced by thiabendazole are mediated via apoptosis, oxidative stress and alteration in PI3K/Akt and MAPK pathways in zebrafish. Environ Int (2023) 176:107973. doi: 10.1016/j.envint.2023.107973 37196567

[35] Paccosi E Balzerano A Proietti-De-Santis L. Interfering with the ubiquitin-mediated regulation of akt as a strategy for cancer treatment. Int J Mol Sci (2023) 24(3):2809. doi: 10.3390/ijms24032809 36769122 PMC9917864

[36] Zhuo DX Zhang XW Jin B Zhang Z Xie BS Wu CL. CSTP1, a novel protein phosphatase, blocks cell cycle, promotes cell apoptosis, and suppresses tumor growth of bladder cancer by directly dephosphorylating Akt at Ser473 site. PloS One (2013) 8(6):e65679. doi: 10.1371/journal.pone.0065679 23799035 PMC3684612

[37] Hanahan D Weinberg RA. The hallmarks of cancer. Cell (2000) 100(1):57–70. doi: 10.1016/S0092-8674(00)81683-9 10647931

[38] Wei Y Zhou J Yu H Jin X. AKT phosphorylation sites of Ser473 and Thr308 regulate AKT degradation. Biosci Biotechnol Biochem (2019) 83(3):429–35. doi: 10.1080/09168451.2018.1549974 30488766

[39] Gallay N Dos Santos C Cuzin L Bousquet M Simmonet Gouy V Chaussade C. The level of AKT phosphorylation on threonine 308 but not on serine 473 is associated with high-risk cytogenetics and predicts poor overall survival in acute myeloid leukaemia. Leukemia (2009) 23(6):1029–38. doi: 10.1038/leu.2008.395 19158829

[40] Jiang N Dai Q Su X Fu J Feng X Peng J. Role of PI3K/AKT pathway in cancer: the framework of Malignant behavior. Mol Biol Rep (2020) 47(6):4587–629. doi: 10.1007/s11033-020-05435-1 PMC7295848 32333246

