# Supplementary figures and images for: LTe2 induces cell apoptosis in multiple myeloma by suppressing AKT phosphorylation at Thr308 and Ser473
Source: Front Oncol. 2023 Sep 14;13:1269670. doi: 10.3389/fonc.2023.1269670 (PMC10539572; doi:10.3389/fonc.2023.1269670)

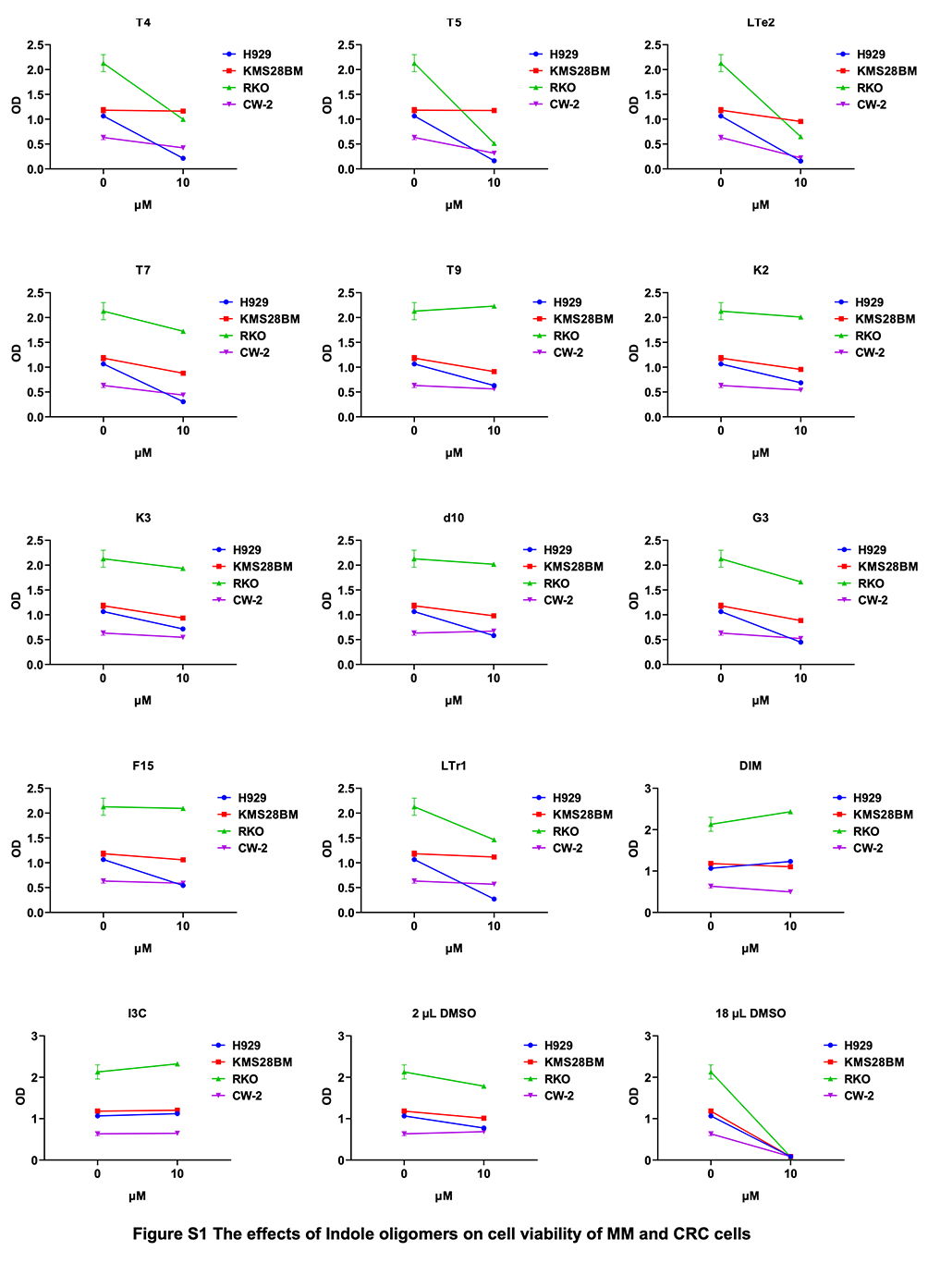

Supplement: Supplementary file 1 [file Image_1.tif]

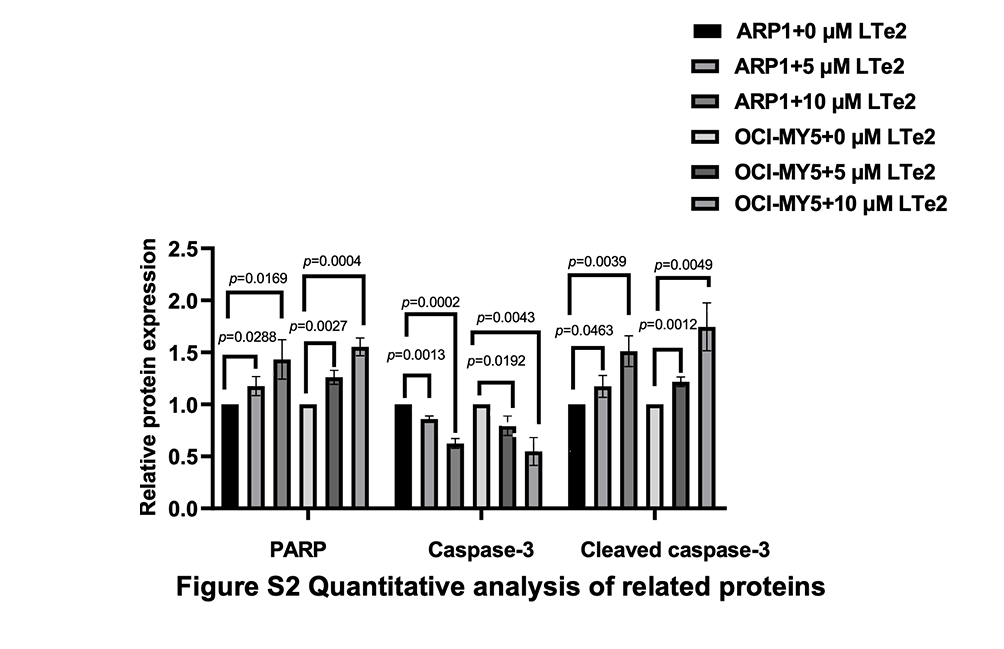

Supplement: Supplementary file 2 [file Image_2.tif]

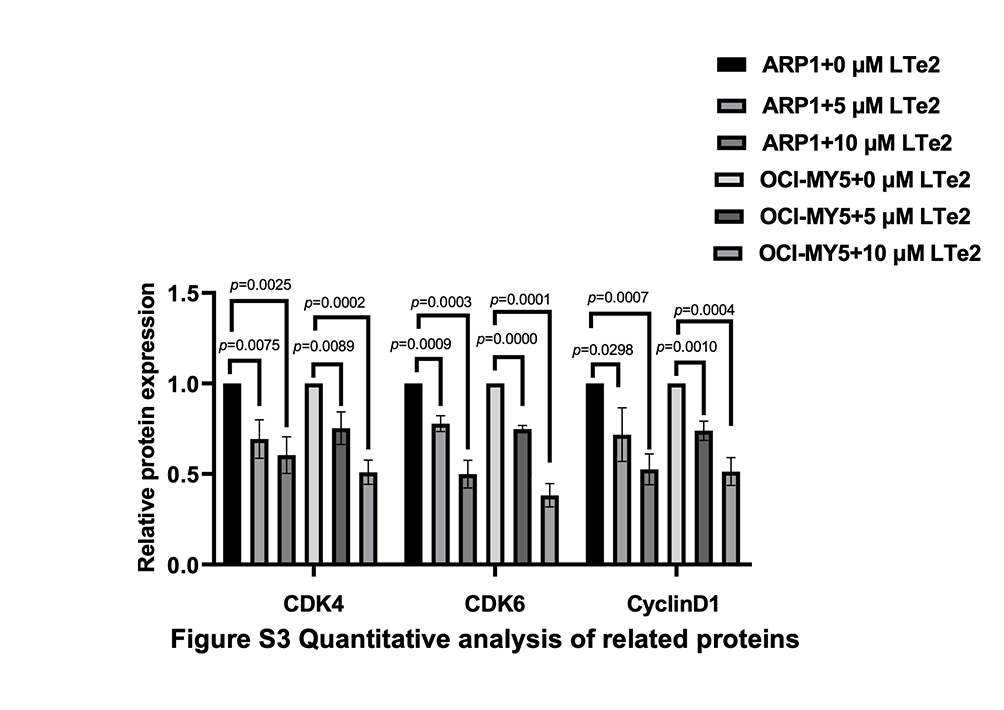

Supplement: Supplementary file 3 [file Image_3.tif]

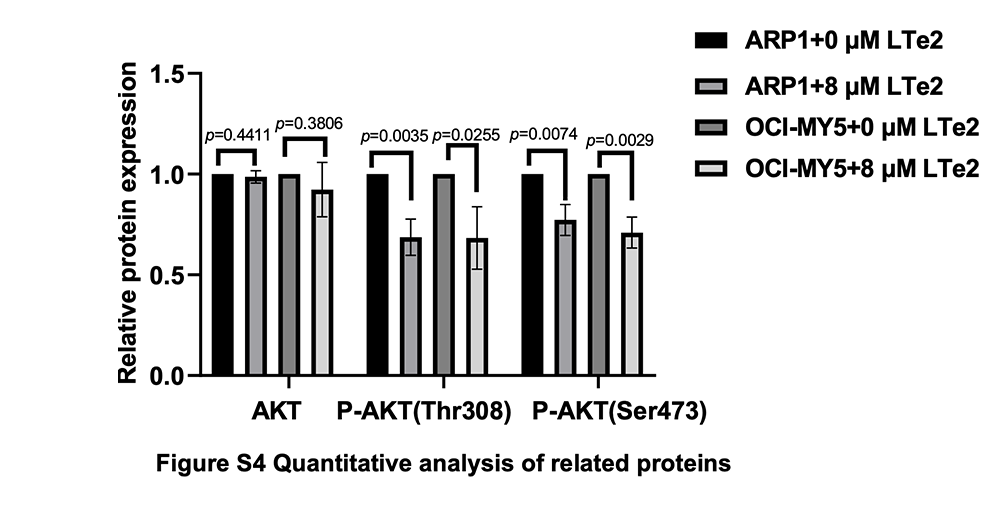

Supplement: Supplementary file 4 [file Image_4.tif]
