# Supplementary material for: LTe2 induces cell apoptosis in multiple myeloma by suppressing AKT phosphorylation at Thr308 and Ser473
Source: Front Oncol. 2023 Sep 14;13:1269670. doi: 10.3389/fonc.2023.1269670 (PMC10539572; doi:10.3389/fonc.2023.1269670)
Supplement: Supplementary file 5 [file Table_1.docx]

**Supplementary table S1 statistics of Pathway Enrichment**

| pathway_id | pathway_name | S gene number | TS gene number | B gene number | TB gene number | pvalue |
| --- | --- | --- | --- | --- | --- | --- |
| ko05168 | Herpes simplex virus 1 infection | 156 | 1546 | 490 | 7775 | 0.00 |
| ko04145 | Phagosome | 46 | 1546 | 148 | 7775 | 0.00 |
| ko04911 | Insulin secretion | 29 | 1546 | 86 | 7775 | 0.00 |
| ko04666 | Fc gamma R-mediated phagocytosis | 30 | 1546 | 90 | 7775 | 0.00 |
| ko04928 | Parathyroid hormone synthesis, secretion and action | 34 | 1546 | 106 | 7775 | 0.00 |
| ko04015 | Rap1 signaling pathway | 58 | 1546 | 206 | 7775 | 0.00 |
| ko04068 | FoxO signaling pathway | 40 | 1546 | 133 | 7775 | 0.00 |
| ko05130 | Pathogenic Escherichia coli infection | 20 | 1546 | 55 | 7775 | 0.00 |
| ko05200 | Pathways in cancer | 130 | 1546 | 528 | 7775 | 0.00 |
| ko05205 | Proteoglycans in cancer | 56 | 1546 | 201 | 7775 | 0.00 |
| ko05164 | Influenza A | 48 | 1546 | 169 | 7775 | 0.00 |
| ko04210 | Apoptosis | 40 | 1546 | 136 | 7775 | 0.00 |
| ko04115 | p53 signaling pathway | 24 | 1546 | 72 | 7775 | 0.00 |
| ko05132 | Salmonella infection | 27 | 1546 | 84 | 7775 | 0.01 |
| ko04540 | Gap junction | 28 | 1546 | 88 | 7775 | 0.01 |
| ko04390 | Hippo signaling pathway | 44 | 1546 | 154 | 7775 | 0.01 |
| ko04064 | NF-kappa B signaling pathway | 29 | 1546 | 93 | 7775 | 0.01 |
| ko05140 | Leishmaniasis | 23 | 1546 | 70 | 7775 | 0.01 |
| ko00562 | Inositol phosphate metabolism | 24 | 1546 | 74 | 7775 | 0.01 |
| ko04010 | MAPK signaling pathway | 76 | 1546 | 295 | 7775 | 0.01 |

S: Significant ID Number; TS: Total Significant ID Number; B: Background ID Number; TB: Total Background ID Number
